# The “Kabedon” technique for treatment of abdominal aortic aneurysm with severe angulation of infrarenal aortic neck

**DOI:** 10.3389/fsurg.2025.1593437

**Published:** 2025-09-08

**Authors:** Xiaozhi Sun, Wenjing Huang, Huibo Ma, Hongyu Qu, Heng Zhang, Junwei Yan, XueFei Jiao, Junjun Liu, Mingjin Guo

**Affiliations:** ^1^ Department of Vascular Surgery, The Affiliated Hospital of Qingdao University, Qingdao, Shandong, China; ^2^ Department of Vascular Surgery, Peking Union Medical College Hospital, Chinese Academy of Medical Sciences, Beijing, China; ^3^ Department of Interventional Medicine and Vascular, Binzhou Medical University Hospital, Yantai, Shandong, China; ^4^ Department of Vascular Surgery, Shanghai Fourth People’s Hospital, School of Medicine, Tongji University, Shanghai, China

**Keywords:** abdominal aortic aneurysm, severe infrarenal neck angulation, endovascular aneurysm repair, Kabedon technique, short-term outcomes

## Abstract

**Objective:**

To evaluate the feasibility and short-term clinical outcomes of the Kabedon technique-based endovascular aneurysm repair (EVAR) for abdominal aortic aneurysms (AAA) with severe infrarenal neck angulation (angle >60°).

**Methods:**

This retrospective cohort study was based on a single-center database. Between January 2019 and May 2023, 120 patients with AAA of hostile neck angulation underwent endovascular procedures using the Kabedon technique for abdominal aortic remodeling. A standardized protocol was followed to calculate the serial changes in the aneurysmal neck angle. The primary endpoints were proximal type Ia endoleak and stent-graft migration. The secondary endpoints were all-cause and aneurysm-related mortality, proximal neck dilatation, and re-intervention.

**Results:**

The mean age was 71.40 ± 10.69 years, and 95 (79.17%) were male. The mean AAA sac diameter and proximal neck angle were 63.71 ± 17.32 mm and 83.67 ± 18.45°, respectively. All patients underwent the Kabedon-based EVAR, with a technical success rate of 94.17% (113/120). During the operation, 7 cases of endoleak and 2 cases of endograft migration were observed, which were resolved by corresponding measures such as coil embolization and proximal cuff stent salvage. No complications were observed within 30 days. In addition, neck calcification, funnel-shaped aneurysm, intraoperative complications and corresponding treatments may be potential negative factors for technical success, but there was no statistical difference.

**Conclusions:**

Kabedon-based EVAR for AAA with a severely angulated neck provided high technical success, low mortality and complication rates during short-term follow-up. Further studies with larger sample sizes and longer follow-up periods are warranted.

## Introduction

Endovascular aneurysm repair (EVAR) has become an effective alternative procedure for open repair of abdominal aortic aneurysm (AAA) owing to a shorter hospital stay and lower postoperative mortality and morbidity (1–4). Upwards of 30% of patients with AAA have unsuitable proximal neck morphology for conventional endovascular repair (5, 6). The most common prognostic factors for poor outcomes include short infrarenal aneurysmal neck, large neck diameter, large aneurysmal sac diameter, neck thrombus, and complex iliac artery anatomy (7–9). Particularly in patients with severe proximal neck angulation (angle >60°), it not only increases the technical difficulty of stent placement but also results in poor short-term outcomes, with a high incidence of type Ia endoleak and graft migration (10–12). Recently, advanced endovascular techniques and devices have been developed to make stent-graft sealing easier by adjusting the neck angulation (13–15). However, procedures using these innovations are still technically demanding and cannot exclude the possibility of endoleaks and migration (16).

In this study, we propose a novel stent-graft deployment technique termed the “Kabedon” technique. Utilizing a stiff guidewire and highly conformable endograft, this method adapts to severe aneurysm neck angulation and conforms to aortic curvature, achieving secure fixation with optimized wall apposition and consequent hemodynamic enhancement. On this basis, we determined the feasibility and short-term clinical outcomes of this novel procedure for AAA with hostile neck anatomy.

## Methods

### Study design and patient selection

This was a single-center retrospective observational study. From January 2019 to May 2023, 120 consecutive patients underwent EVAR using the Kabedon technique at the Affiliated Hospital of Qingdao University, Shandong, China. They were diagnosed as having AAA with severe proximal neck angulation, by computer tomography angiography (CTA). The inclusion criteria for this study were as follows: (1) infrarenal aortic angulation >60°; (2) proximal neck length >15 mm; (3) distal iliac fixation site diameter <16 mm and >30 mm in length (17); (4) life expectancy >1 years exclusion criteria were as follows: (1) low operative risk for open repair, (2) connective tissue disease, (3) ASA grade IV or V, (4) proximal neck thrombus >50% of circumference, (5) proximal neck calcification >50% of circumference (18); (6) Infected AAA.

**Figure 1:**
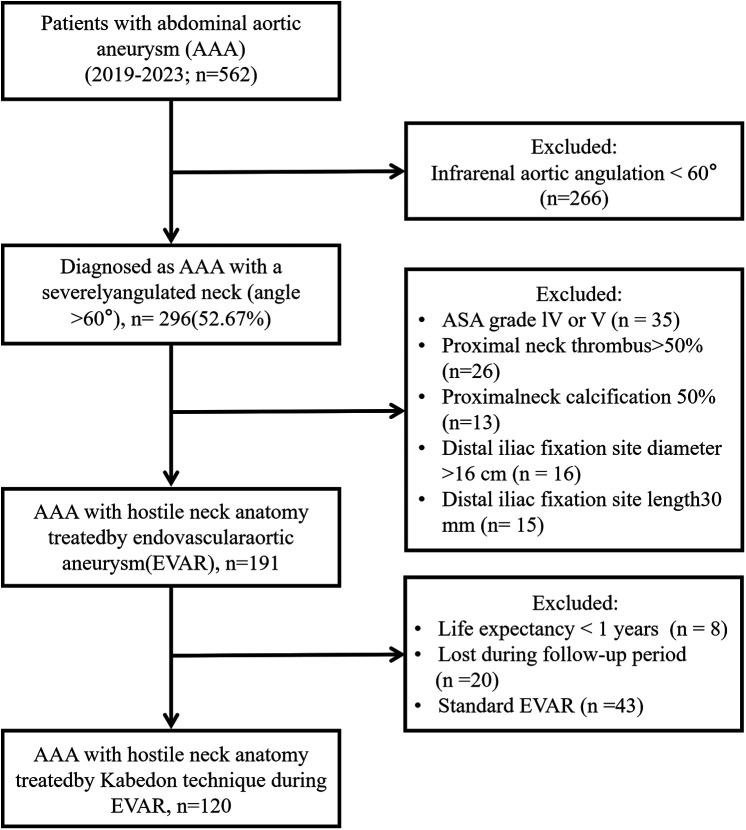
Flow diagram of the enrolled patients of abdominal aortic aneurysm with a severely angulated neck (angle >60°) by Kabedon technique during endovascular aortic repair.

Patient demographics, risk factors, operational details, and clinical outcomes were also recorded. Aortic morphology data were obtained using preoperative CTA. The study protocol was approved by the local institutional review board and Ethics Committee (Figure 1).

### Pre-operative evaluation

Two experienced vascular surgeons used a standardized CT measurement protocol to calculate the serial change in AAA neck angulation using three-dimensional (3D) volume-rendered images generated via post-acquisition software (Endosize, TherenvaSAS, France) before the procedure. The AAA characteristics recorded were the maximum diameter of the AAA and proximal neck anatomy, including diameter, length, angulation, thrombus, and calcification. A center lumen line (CLL) was used to measure diameter and length. The maximum aneurysm diameter and AAA neck length were measured in the axial view (1.0-mm slices) perpendicular to just below the most caudal renal artery. Similarly, the proximal neck angle was measured as described by Chaikof et al. Calcification and thrombus were measured at the level of the lowest renal artery (19). The aortic neck scoring system, according to the Society for Vascular Surgery grading classification for morphological risk, was used.

### Selection of endovascular stent grafts

Aortoiliac sizing and endograft planning were performed according to our routine preoperative evaluation practice (20). The selection of aortic stent graft, iliac artery stent, and stiff guidewire type was based on institutional practice and surgeon experience, as were other technical aspects of EVAR procedures performed.

### “Kabedon” technique in endovascular procedure

**Figure 2:**
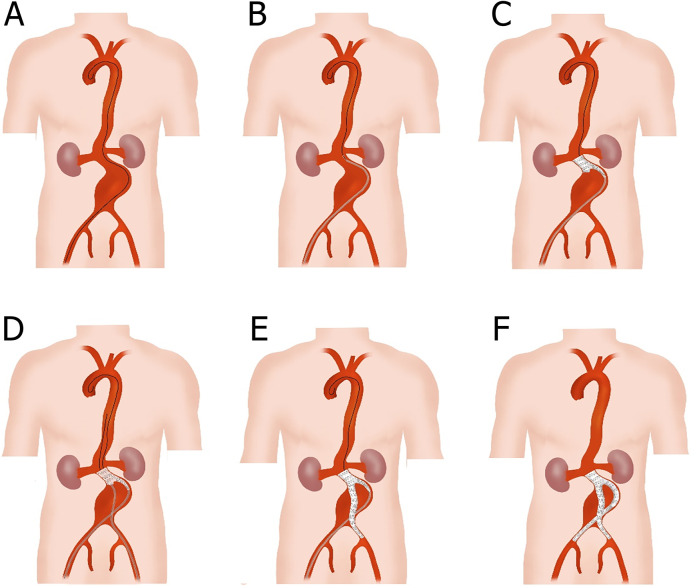
Schematic diagram of the total endovascular treatment with Kabedon technique for abdominal aortic aneurysm with severely angulated neck. **(A)** Exchange the ultra-sliding guidewire with the stiff guidewire; then, along the large curvature of the AAA, the top end of the stiff guidewire was advanced to the aortic valve and formed a loop. **(B)** A bifurcated main-body stent graft was placed at the lower edge of the lowest renal artery with the aid of a delivery sheath. **(C)** The main body of the stent graft was deployed without opening its long limb. **(D)** Another exchanged stiff guidewire and delivery sheath were advanced to the short limb of the main body stent graft through the contralateral femoral artery. **(E)** The contralateral common iliac artery was reconstructed with a branch membrane-covered stent, and the end of the branch stent was positioned at the upper edge of the internal iliac artery. **(F)** The ipsilateral common iliac artery was also reconstructed using a branch membrane-covered stent, and a cross-limb structure was eventually formed.

**Figure 3:**
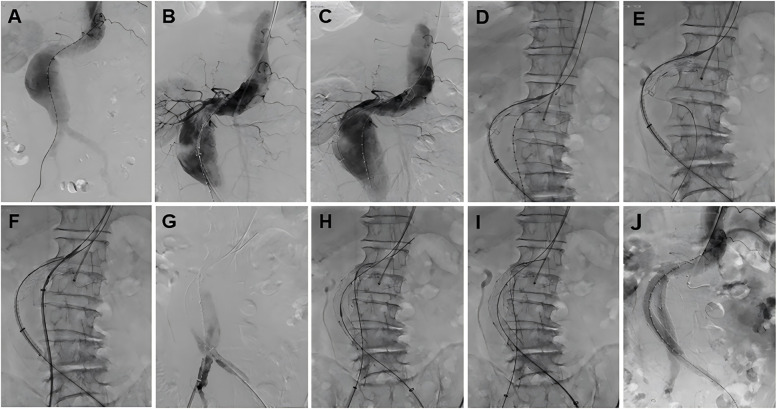
The fluoroscopy procedural steps of the total endovascular treatment with Kabedon technique for treatment of abdominal aortic aneurysm with severely angulated neck. **(A)** Abdominal aortography. **(B)** Access the stent to identify the position of the renal arteries. **(C)** The guidewire provides tension, and the stent delivery system fits the aneurysm. **(D)** Deploy the stent while maintaining guidewire tension. **(E)** Select the contralateral iliac branch. **(F)** Deliver the right iliac artery stent system. **(G)** Identify the bifurcation position of the right common iliac artery. **(H)** Deploy the right iliac limb. **(I)** Deploy the left iliac limb. **(J)** Angiography after stent deployment. From **(D–I)**, the guidewire is required to provide tension to make the stent fit the aneurysm perfectly, and finally, the iliac limbs on both sides need to reliably provide subsequent stability.

The term “Kabedon” is derived from Japanese popular culture, describing the act of confining someone against a wall with one arm. This metaphorically reflects the technique's core principle: the stiff guidewire forms a supportive “arm” along the aortic curvature (Figure 2B), confining the stent-graft against the greater curvature wall to optimize sealing.All procedures were performed under general anesthesia by experienced vascular surgeons in a hybrid vascular operative room. Systemic heparinization of 100 IU/kg was performed after the percutaneous arterial puncture. The femoral artery was cannulated with an 8 Fr sheath, and a 0.035-inch ultra-sliding guidewire and 5F pigtail catheter were placed. Initial anteroposterior abdominal aortography was performed to inspect the location of the renal or iliac arteries, and the hostile anatomy was characterized by a severely angulated proximal neck. The stiff guidewire was advanced into the ascending aorta to form the “Loop,” then it was kept in tension to establish a through-and-through guidewire along the large curvature of AAA. The stiff guidewire was called “Kabedon.” When the highly conformable endograft was in position, we imposed a certain tension on the through-and-through guidewire to create a stent graft along the aortic curvature, obtaining the ideal aortic neck–endograft alignment. Bilateral iliac arteries were reconstructed using a cross-limb configuration (21). If an iliac aneurysm was observed, concomitant IIA embolization was performed using detachable coils before the reconstruction of the iliac arteries. Final angiography was performed to verify the correct position of the endograft and the absence of an endoleak (Figures 2, 3).

### Perioperative monitor and follow-up

Aspirin (100 mg) was administered starting at 1-day post-surgery. Perioperative characteristics were recorded, including adjunctive procedures, procedure and fluoroscopy times, contrast volume, primary technical success, and days of hospitalization. Technical success was defined as the successful introduction and deployment of a stent graft in the absence of stent graft migration, exclusion of the aneurysmal sac on the completion angiogram, and freedom from type Ia or III endoleak or graft limb occlusion. Follow-up visits involved clinical examination and aortic CT angiography (CTA) and were performed at 1, 6, and 12 months and annually after discharge, followed by duplex ultrasound. The clinical course (mortality, morbidity, and incidence of post-implantation syndrome) and follow-up CTA (proximal neck dilatation, endoleak, graft thrombosis, migration, and rupture) were also recorded for each patient. The primary endpoints were proximal type Ia endoleak and stent graft migration. The secondary endpoints were all-cause and aneurysm-related mortality, proximal neck dilatation, aneurysm rupture, and re-intervention.

### Statistical analysis

All statistical analyses were performed using the IBM SPSS software (version 23.0; IBM Corp., Armonk, NY, USA). Continuous variables are described as mean ± standard deviation (SD), and the specific data are presented as counts and percentages. For continuous variables, the Student's *t*-test was conducted if the variable was normally distributed, whereas the Mann–Whitney *U*-test was used for non-normally distributed variables. The chi-squared test was used for categorical variables. Additionally, logistic regression was employed to identify variables potentially associated with the study endpoint. Statistical *p*-value < 0.05.

## Results

### Baseline clinical characteristics

The clinical and anatomical baseline characteristics are presented in Table 1. 120 patients with AAA and severe neck angulation were evaluated for Kabedon-based EVAR at our center. The mean age was 71.40 ± 10.69 years, and 79.17% were male. The top three comorbidities were hypertension (*n* = 82, 68.33%), smoking history (*n* = 90, 75.00%), and coronary artery disease (*n* = 46, 38.33%).

**Table 1:** Baseline clinical characteristics.

Variables	Patients (*n* = 120)
Demographics	
Age, years	71.40 ± 10.69
Male, *n* (%)	95 (79.17)
Medications	
Antiplatelet agents use	115 (95.83)
Statins use	65 (54.17)
Comorbidities	
Hypertension, *n* (%)	82 (68.33)
Hyperlipidemia, *n* (%)	58 (48.33)
Smoking history, *n* (%)	90 (75.00)
Coronary artery disease, *n* (%)	46 (38.33)
Diabetes mellitus, *n* (%)	23 (19.17)
COPD, *n* (%)	37 (30.84)
CKD (Cr > 2 mg/dl), *n* (%)	5 (4.27)
Cerebrovascular disease, *n* (%)	7 (5.83)
Peripheral artery disease, *n* (%)	49 (40.83)

COPD, chronic obstructive pulmonary disease; CKD, chronic kidney disease. Continuous data are presented as mean ± standard deviation, and qualitative data are presented as numbers and percentages.

### Preoperative morphological characteristic

The mean maximum diameter of AAA was 63.71 ± 17.32 mm, with a mean aneurysm volume of 310.37 ± 20.51 cc and proximal neck angle of 83.67 ± 18.45°. The neck angulation was >60° in all cases (100.00%) and ≥90° in 53 (44.17%). The mean proximal neck length was 39.50 ± 26.28 mm, and the mean proximal neck diameter was 24.90 ± 8.97 mm. 25 (20.84%) and 43 patients (35.83%) were respectively observed with neck thrombus and calcification >25% of the circumference. 64 patients (53.33%) had aortic neck scores were >3 points. The rate of aneurysm at the distal iliac artery was 40.00% (48/120). In terms of AAA shape, most are Straight or Tapered, but there are still 9 cases (7.50%) showing Funnel. All the data are presented in Table 2.

**Table 2:** Preoperative morphological characteristics.

Variables	Patients (*n* = 120)
Maximum AAA diameter, mm	63.71 ± 17.32
AAA sac volume, cc	310.37 ± 20.51
Proximal neck abnormalities	
Proximal neck angle	
Mean (°)	83.67 ± 18.45
≥90°	53 (44.17)
60°–90°	67 (55.83)
Proximal neck diameter, mm	24.90 ± 8.97
Proximal neck Length, mm	39.50 ± 26.28
Neck thrombus >25% of circumference (*n*)	43 (35.83)
Neck calcification >25% of circumference (*n*)	25 (20.84)
Aortic neck score >3	64 (53.33)
Distal iliac artery abnormalities	
Aneurysm	48 (40.00)
Shape	
Straight	77 (64.17)
Dumbbell	10 (8.33)
Tapered	24 (20.000)
Funnel	9 (7.50)

AAA, abdominal aortic aneurysm.

Continuous data are presented as mean ± standard deviation, and qualitative data are presented as numbers and percentages.

### Details and outcomes of the surgery

The intraoperative details of the 120 patients treated with Kabedon-based EVAR are presented in Table 3. Among all patients, 16 (13.33%) underwent emergency EVAR due to unstable AAA. The overall operational time was 105.00 ± 30.13 min. The mean contrast volume and fluoroscopic time were 97.31 ± 22.44 ml and 30.25 ± 4.31 min. The median oversize of the proximal aorta was 21.42 ± 8.55%. The mean diameter of the aortic stent graft was 28.12 ± 11.33 mm. The mean centerline length of the stent graft in the proximal landing zone was 36.21 ± 14.27 mm and 16.78 ± 12.13 mm in the modeled and actual seal zones, respectively. Comfortable aortic stent grafts were used to reconstruct the AAA, including those using Excluder (Gore C3, *n* = 87) and Minos (MicroPort, *n* = 33). The bridge endograft for the common iliac artery was the Iliac Branch Endoprosthesis of corresponding brands (W.L. Gore & Associates, USA, *n* = 87; (Shanghai MicroPort Endovascular MedTech Co., Shanghai, PRC, *n* = 33). In terms of surgical outcomes, the technical success rate reached 94.17% (113/120). 2 (1.67%) patients required conversion to open surgery due to AAA rupture, and 1 (0.83%) patient with ruptured AAA died intraoperatively due to hemorrhagic shock. Regarding complications, 7 (5.83%) patients experienced immediate endograft migration and endoleak formation. They underwent coil embolization and proximal cuff stent bridging. A patients received chimney procedure for renal artery reconstruction.

**Table 3:** Operative procedures of 120 patients treated by Kabedon-based endovascular aneurysm repair for abdominal aortic aneurysm with a severely angulated neck.

Variables	Patients (*n* = 150)
Emergency surgery	16 (13.33)
Anesthesia methods	
General anesthesia	67 (55.83)
Combined anesthesia	53 (44.17)
Operation time (min)	105.00 ± 30.13
Contrast volume (ml)	97.31 ± 22.44
Fluoroscopic time (min)	30.25 ± 4.31
Oversize, %	21.42 ± 8.55
Proximal sealing zone, mm	
Mean diameter, mm	28.12 ± 11.33
Mean length	
Modeled seal zone centerline length, mm	36.21 ± 14.27
Actual seal zone centerline length, mm	16.78 ± 12.13
Distal sealing zone, cm	5.37 ± 16.12
Stiff guidewire Brand	
Amplatz Super Stiff (Cordis Crop.)	115 (95.83)
Lunderquist (COOK, USA)	5 (4.17)
Reconstruction of the abdominal aortic aneurysm	
Diameter, mm	34.67 ± 14.12
Brand	
Gore Excluder (W.L. Gore & Associates, Flagstaff, Ariz)	87 (72.50)
Minos (Shanghai MicroPort Endovascular MedTech Co., Shanghai, PRC)	33 (27.50)
Reconstruction of the common iliac artery	
Diameter, mm	13.87 ± 3.71
Length, cm	13.21 ± 2.08
Brand	
Iiiac Branch Endoprosthesis (W.L. Gore & Associates, USA)	120 (100.000)
Complications	
Endoleak	7 (5.83)
Endograft migration	2 (1.67)
Coil embolization	5 (4.17)
Proximal CUFF stent salvage	4 (3.33)
Surgical outcomes	
Technical success^a^	113 (94.17)
Conversion to open surgery	2 (1.67)
Death	1 (0.83)

Continuous data are presented as mean ± standard deviation, and qualitative data are presented as numbers and percentages.

^a^ Technical success was defined as the ability to adequately deploy the endograft in the intended position, and no type Ia endoleak occurred at the end of the surgery.

### Risk factors of technical success in Kabedon-EVAR

To evaluate the application value of the Kabedon technique, we analyzed the factors that may be related to its technical success rate. Univariate analysis showed that the shape of the aneurysm was associated with technical success, with patients having funnel-shaped aneurysms showing a lower technical success rate. In addition, patients with neck calcification were associated with technical failure. From the details of the surgery, complications such as endoleaks and endograft migration occurring during the operation, as well as procedures like coil embolization and proximal CUFF stent salvage, may all indicate a lower technical success rate. According to the research results, no other factors showed a potential correlation with the technical success rate (Table 4).

**Table 4:** Univariate analysis of risk factors for technical success in Kabedon-EVAR.

Variables	Technical success (*n* = 113)	Unsuccess (*n* = 7)	*P*-value
Age, years	71.66 ± 8.81	71.00 ± 7.66	0.846
Male	89 (78.76)	6 (85.71)	0.660
BMI (kg/m^2^)	23.84 ± 4.36	24.48 ± 2.89	0.701
Hypertension	77 (68.14)	5 (71.43)	0.856
Hyperlipidemia	33 (24.50)	2 (28.57)	0.972
Smoking history	67 (59.29)	3 (42.86)	0.392
Coronary artery disease	52 (46.02)	4 (57.14)	0.567
Diabetes mellitus	27 (22.50)	1 (14.29)	0.560
COPD	4 (3.54)	0 (0.00)	0.613
CKD	10 (8.85)	0 (0.00)	0.411
Cerebrovascular disease	19 (16.81)	0 (0.00)	0.237
Maximum AAA diameter, mm	60.94 ± 17.10	65.00 ± 20.41	0.547
Proximal neck angle	82.32 ± 17.20	82.34 ± 22.60	0.997
Proximal neck diameter	23.99 ± 6.50	29.00 ± 15.11	0.076
Proximal neck Length	41.12 ± 24.75	48.30 ± 21.34	0.456
Neck thrombus >25% of circumference	23 (20.35)	2 (28.57)	0.603
Neck calcification >25% of circumference	34 (30.09)	7 (100.00)	0.001*
Iliac artery aneurysm	44 (38.94)	4 (57.14)	0.340
Funnel aneurysm	2 (1.77)	7 (100.00)	0.001*
Emergency surgery	15 (13.27)	1 (14.29)	0.939
General anesthesia	62 (54.87)	5 (71.43)	0.392
Operation time (min)	88.79 ± 38.12	78.57 ± 26.25	0.487
Endoleak	5 (4.42)	2 (28.57)	0.008*
Endograft migration	0 (0.00)	2 (28.57)	0.001*
Coil embolization	1 (0.88)	4 (57.14)	0.001*
Proximal CUFF stent salvage	0 (0.00)	4 (57.14)	0.001*

Continuous data are presented as mean ± standard deviation; qualitative data are expressed as median and minimum-maximum range or number and percentage.

*Significant difference.

Based on the results of the univariate analysis and clinical relevance, we selected 6 variables for multivariate regression analysis. Although all the aforementioned factors suggested that they might be negative influencing factors of the Kabedon technique, the current data analysis indicated that there were no statistically significant differences (Table 5).

**Table 5:** Factors associated with technical success on multivariable regression analysis.

Variables	B	Exp (B)	*P*-value
Neck calcification >25% of circumference	−1.372	0.254	0.984
Funnel aneurysm	−33.042	0.001	0.716
Endoleak	−32.394	0.001	0.994
Endograft migration	−33.214	0.001	0.994
Coil embolization	−33.406	0.001	0.996
Proximal CUFF stent salvage	−37.124	0.001	0.998

### Early and short-term follow-up outcomes

**Figure 4:**
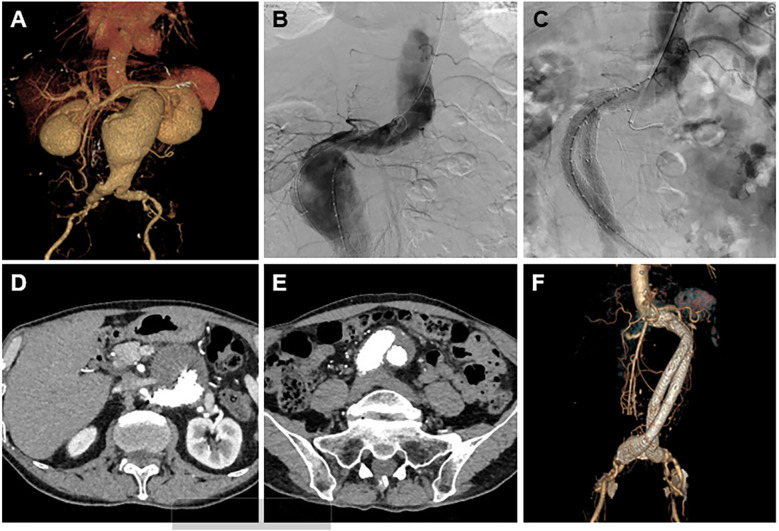
A result of the treatment and follow-up using the Kabedon technique for endovascular management of severely angulated neck infrarenal abdominal aortic aneurysms. **(A)** Preoperative CTA 3-dimensional volume-rendering reconstruction showing a proximally angulated neck, infrarenal aneurysmal neck, and abdominal aortic aneurysm sac. **(B)** Intraoperative angiography showing the stiff guidewire status, herein called “Kabedon.” Tension was maintained through the guidewire along the large curvature of the AAA. **(C)** Completion angiography demonstrates abdominal aorta and limb reconstruction without endoleaks. **(D**,**E)** The latest follow-up CTA showed no endoleaks, and migration occurred at the proximal and distal stent grafts. **(F)** A CTA scan 1 year after endovascular repair showing exclusion of the aneurysmal sac with no evidence of type Ia or III endoleak or graft limb occlusion.

The early and short-term outcomes are summarized in Table 6. The duration of hospitalization was 13.50 ± 4.03 days. In the one-month follow-up, the patients were in stable condition, and no one developed complications such as endograft migration or endoleak. The mean follow-up was 14.00 ± 2.59 months. None of the patients were lost to follow-up. During the follow-up period, 5 patients (4.17%) were found to have sac expansion, among whom 2 (1.67%) had type Ib endoleaks and received active treatment. One (0.83%) patient developed iliac limb occlusion, which was considered possibly due to thrombosis (Table 6). No cases of significant endograft migration were detected in the latest follow-up CTA. The complete imaging treatment and follow-up periods are shown in Figure 4.

**Table 6:** Early and short-term clinical outcomes in 120 cases of abdominal aortic aneurysm with a severely angulated neck treated with Kabedon-based endovascular aneurysm repair.

Outcomes	Patients (*n* = 120)
Hospitalization, days	13.50 ± 4.03
Early outcomes	
30-day mortality	0 (0.00)
Endograft migration	0 (0.00)
Endoleaks	
Type Ia	0 (0.00)
Type Ib	0 (0.00)
Type II	0 (0.00)
Type III	0 (0.00)
Short-term outcomes	
Median Follow-up time, months	14.00 ± 2.59
All-cause mortality	4 (0.00)
AAA-related adverse events*	
Endoleaks	
Type Ia	0 (0.00)
Type Ib	3 (2.50)
Type II	4 (3.33)
Type III	1 (0.83)
Proximal neck dilatation	0 (0.00)
Aneurysm rupture	0 (0.00)
Limb occlusion	1 (4.0)
Endograft migration	0 (0.00)
Sac expansion	5 (4.17)
Reintervention	5 (4.17)

Continuous data are presented as mean ± standard deviation; qualitative data are expressed as median and minimum-maximum range or number and percentage.

AAA-related adverse events include higher luminal volumes and wider, shorter, and more angulated proximal neck.

*Significant difference.

## Discussion

Since the first report of EVAR by Parodi et al. in 1991, this procedure has been introduced as an alternative to open surgery, although it has become a popular treatment associated with low perioperative mortality and short hospital stays for AAA patients (22).

However, the incidence rate of complications and overall and aneurysm-related mortality in the long term is higher after EVAR than after open surgical repair (23). Adverse morphological features of the proximal aortic neck are considered major factors for EVAR failure related to proximal endoleaks and migration (24). In particular, a severely angulated proximal aortic neck is associated with an increased risk of adverse aneurysm-related events (25). Currently, there is no consensus on the optimal management of AAA with a severely angulated neck during EVAR.

Currently, the main solutions for tortuous aortic necks primarily involve applying external forces to correct the tortuous angle. One approach is to use an ultra-stiff guidewire to correct the tortuous neck before stent deployment. However, the tension generated after forced correction of the neck may cause stent migration once the deployment guidewire is withdrawn. Moreover, the stent struggles to resist neck retraction, resulting in unstable fit between the stent and the arterial wall, which can lead to endoleaks. Taneva et al. reported a multicenter study showing that the incidence rates of early and late type I endoleaks using this modified technique could reach 16.4% and 12.1%, respectively (26). In addition, placing a CUFF stent at the neck first can correct the neck angulation to a certain extent, increase the effective length of the neck to obtain a longer anchoring zone, and allow better fixation of the main stent. Similarly, this technique still cannot completely relieve the stress of the stent on the aortic wall, which can easily lead to complications such as stent migration, endoleak, and occlusion. Hobo et al. found that the incidence rates of early and late type I endoleaks after applying this technique were 3.2%–6.5%, and the incidence rate of stent migration was 4.3%–5.9% (27, 28).

In the present study, we report a single-center experience of 120 consecutive patients with hostile anatomy of the proximal neck managed by the novel Kabedon technique. It is a novel technique for complying with aneurysmal morphology, with a flexible proximal configuration and proximal active control system to fit a severely angulated neck. According to our experience, in a severely angulated neck, the initial deployment of the endograft should be performed above the lowest renal artery ostium to reposition the endograft by pulling it downstream rather than pushing it cranially. In short, the shape of the neck can be maximized to increase the contact area between the stent and artery wall.

However, stent-graft stiffness is recognized as another major risk factor for early type Ia endoleaks and is caused by small gaps formed between the severely angulated neck and proximal stent-graft (11). Greater graft oversizing is needed to seal the proximal attachment zone, leading to potential graft migration associated with neck dilation due to negative remodeling over time (29). Therefore, a highly comfortable aortic endograft combined with a mean oversize effectively decreased this gap effectively and not have negative effects on aortic dilatation. Given that the present series had a high prevalence of more severe neck angulation on pre-EVAR CTA, the primary technical success rate was still excellent, which may be related to an increase in neck length via the tension of stiff guidewire and the enhanced anchoring effect of the stent-grafts, preventing acute endograft migration during the procedure.

Generally, the Amplatz guidewire is used to establish the “Kabedon” pathway. Compared to the Amplatz (Boston Scientific, Hemel Hempstead, UK) guidewire, the Lunderquist (Cook Aortic Interventions, Bloomington, USA) guidewire has a stiffer body and is not easy to bend (30). It may have resulted in the rebound of the stent-grafts, and the neck was forced to straighten the aneurysm morphology and then cause upward displacement of the stent-grafts. Once the stent-grafts is released, it is difficult to change its release position. A patient who used the Lunderquist guidewire experienced upward displacement of the stent-grafts. Chimney grafts were released to remedy the double renal arteries and prevent the renal arteries from being covered, and no adverse events occurred after the operation.

Distal attachment sites are of great importance for the feasibility and durability of EVAR. The “hypogastric snorkel” technique similarly involves the placement of a covered stent-grafts parallel to an iliac limb device to preserve perfusion to the hypogastric artery to extend the distal sealing zone in short common iliac arteries or cases of common iliac artery aneurysms (31). However, Wang et al. reported that the cross-limb procedure in EVAR was more advantageous than the standard limb configuration, particularly in patients with large aneurysm sacs or tortuous iliac arteries. The Pericles registry suggested that type Ia endoleaks can be minimized by utilizing at least 20 mm of the landing zone (32). In this study, bilateral iliac arteries were reconstructed using a cross-limb configuration to increase the distal sealing zone.

In this study, we observed that complications such as neck calcification, funnel-shaped aneurysm sac, type I endoleak, and procedures like proximal cuff stent salvage may all be negative predictors of the success of the Kabedon-EVAR technique. Interestingly, these multiple factors actually represent the potential dilemmas encountered during surgery for aneurysms with complex necks. Calcified necks and irregular aneurysm bodies have poor compliance, leading to inadequate adherence and greater difficulty in stent fixation, which increases the likelihood of endograft migration and type I endoleak. For this reason, remedial measures such as coil embolization and proximal cuff stent salvage need to be adopted. Therefore, the degree of neck calcification and the morphology of the aneurysm body should be features of key concern. Although there was no statistical difference in the multivariate analysis, this may be related to the small number of positive outcomes, and longer follow-up and further analysis are required.

The limitations of this study are its small sample size and the fact that only two brands of aortic stent graft device were used. In addition, we retrospectively analyzed the outcomes of EVAR using one technique (the Kabedon technique) and did not directly compare the outcomes of several techniques. Finally, multicenter, prospective, and large-sized studies with long-term follow-up are warranted to fully gauge the effects of Kabedon-based EVAR for AAA with infrarenal neck angulation.

## Conclusion

Kabedon-based EVAR provided a high technical success rate and no mortality or complication rates during short-term follow-up for AAA with a severely hostile neck. This technique appears to contribute to enhanced technical and clinical success rates, even when the neck angle is more hostile. Future studies with long-term follow-up and larger sample sizes are required to evaluate the durability and risk of the late complications associated with this technique.

## Data Availability

The raw data supporting the conclusions of this article will be made available by the authors, without undue reservation.
